# Mental Health Apps in China: Analysis and Quality Assessment

**DOI:** 10.2196/13236

**Published:** 2019-11-07

**Authors:** Jie Shang, Shaoming Wei, Jianbo Jin, Puhong Zhang

**Affiliations:** 1 The George Institute for Global Health Peking University Health Science Center Beijing China; 2 School of Public Health Peking University Health Science Center Beijing China

**Keywords:** mental health, mental disorder, quality assessment, mobile health, digital health, innovative health, smartphone application

## Abstract

**Background:**

Mental disorders have been a great burden on health care systems, affecting the quality of life of millions of people worldwide. Developing countries, including China, suffer from the double burden of both the increasing mental health issues in population and the deficiency in mental health care resources. The use of mobile health technologies, especially for mobile phone apps, can be a possible solution.

**Objective:**

This review aimed to describe the features and assess the quality of mental health apps in major mobile phone app markets in China and further discuss the priorities for mental health app development.

**Methods:**

Keywords including *psychology*, *psychological health*, *psychological hygiene*, *psychological health service(s)*, *mental*, *mental health*, *mental hygiene*, *mental health service(s)*, *depression*, and *anxiety* were searched in Chinese in 3 Android app markets (*Baidu Mobile Assistant*, *Tencent MyApp*, and *360 Mobile Assistant*) and iOS App Store independently. Mental health apps were then selected according to established criteria for in-depth analysis and quality assessment by the Mobile App Rating Scale.

**Results:**

In total, 63 of 997 mental health apps were analyzed in depth, of which 78% (49/63) were developed by commercial entities for general population, 17% (11/63) were for patients or clients of specialized psychiatric hospitals or counseling agencies, 3% (2/63) were by government or local Centers for Disease Control and Prevention for general information, and 2% (1/63) for students of a university. Major built-in features of the apps included counseling services, mental health education, and self-assessment of mental health status by validated self-rating scales. The overall quality score of the MH apps was *acceptable.*

**Conclusions:**

Mental health apps are emerging in the area of mobile health in China. Popular mental health apps usually provide a synthetic platform organizing resources of information, knowledge, counseling services, self-tests, and management for the general population with mental health-related inquiries. The quality of the apps was rated as *acceptable* on average, suggesting some space for improvement. Official guidelines and regulations are urgently required for the field in the future.

## Introduction

### Background

The global burden of mental disorders has been estimated to account for 32.4% of years lived with disability and 13.0% of disability-adjusted life years [1]. The global lifetime prevalence of any mental disorder ranges from 47.4% in the United States to 12% in Nigeria [2]. In China, the lifetime prevalence of any mental disorder is 16.6% [3].

Challenges, however, are critical for mental health care in China. Clinical resources are limited, but the service demands are increasing fast for mental health–related inquiries, including anxiety and depression [4-6]. The lifetime contact rate of patients with common mental disorders, such as depression, with any mental health care was around 2.7% in Beijing and 3.1% in Shanghai, which was strikingly low even in major metropolitan areas, whereas for the treated patients with any type of mental disorder, only 37.5% measured up to the minimum treatment adequacy [7]. The gap in human resources is also alarming, given that there are 1.7 clinical psychiatrists per 100,000 people in China, compared with 12 to 15 per 100,000 people in countries such as Australia and the United States [7,8]. The mental health resource distribution is also uneven, as most mental health professionals and mental health care facilities are concentrated in the Eastern China, having the more developed regions [9]. Although the management of severe mental disorders has been included in the basic public health service (BPHS) package by the Chinese government, the workload of frontline community health care workers is critical [10,11]. In addition, mental disorders are still strongly stigmatized, and people are reluctant to seek professional help [12-15]. Supportive social environment is also lacking for effective mental health education and early intervention [16]. Therefore, it is urgent to improve the access and delivery of mental health services and reallocate the resources in China.

### Objective

On the other hand, driven essentially by the development of novel technology and the demands of mental health services, mobile phone apps have given birth to the era of digitalized mental health care globally [17-20]. Mental health apps also expand exponentially, especially in developing countries [21-23]. China has a gigantic and growing market of both internet and mobile phone usage [24]. By the end of 2017, there were 753 million Chinese people connected to the internet through the mobile phone, accounting for 97.5% of all internet users in the country [24]. Recently, fruitful research has been done exploring contents and features of mobile phone apps in mental health–related areas globally, for example, smoking cessation, suicide prevention, and bipolar disorder treatment [25-28]. There is a study investigating the features and characteristics of apps on maternal and child health in China [29]. However, no academic literature discussing mental health–relevant apps in China was identified.

Therefore, this study aimed to investigate the features and further evaluate the quality of mental health apps in major mobile phone app markets in China and further discuss the priorities for mental health app development in the future.

## Methods

### Overall Procedures

We adopted the following 4 steps to identify relevant mental health apps and analyze the data: preselection, selection, data extraction, and app quality evaluation. All the 4 steps or procedures were conducted by 2 independent investigators following established criteria and procedures. Any discrepancy that occurred was resolved through discussion under the supervision of a senior researcher.

### Preselection of Mental Health Apps

We chose the top 3 Android app markets, *Baidu Mobile Assistant*, *Tencent MyApp*, and *360 Mobile Assistant*, with the biggest market shares in China, and iOS App Store to identify mental health apps [30].

**Figure 1:**
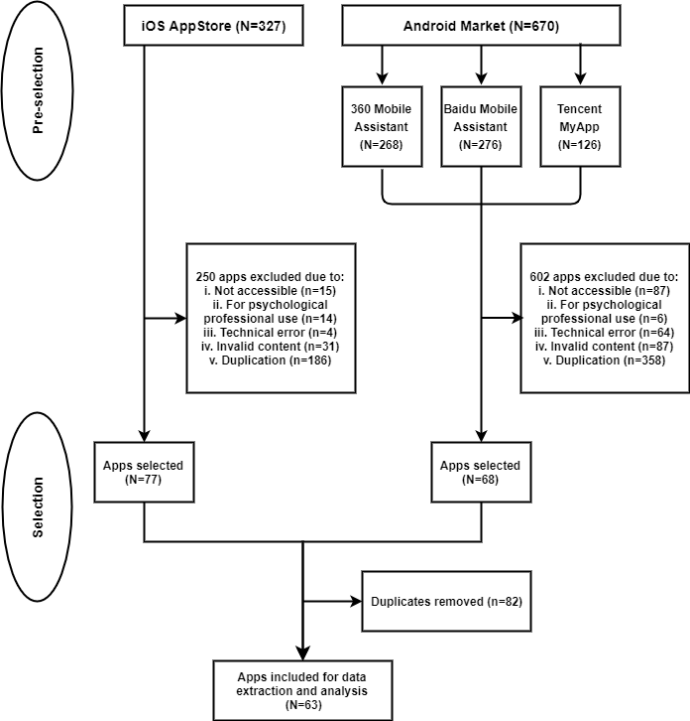
Flow diagram of the preselection and selection procedures of mental health apps.

Keywords *psychology (
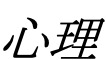
)*, *psychological health (
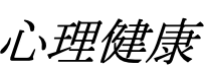
)*, *psychological hygiene (
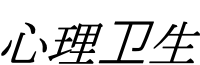
)*, *psychological health service(s) (
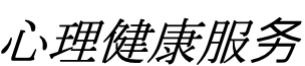
)*, *mental (
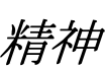
)*, *mental health (
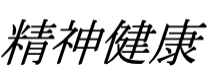
)*, *mental hygiene (
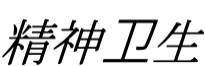
)*, *mental health service(s) (
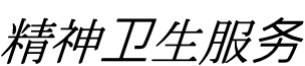
)*, *depression (
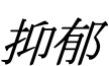
)*, and *anxiety (
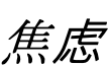
)* were searched in mandarin Chinese on May 14, 2019. Mental health apps were preselected using the following process: (1) for each keyword, the top 50 apps (if available) appeared were reviewed based on their names and app description to identify the relevant apps; and (2) for each app market, all the relevant apps under the 10 keywords were collected, which gave the number of preselected apps. Apps preselected from the 3 Android markets were combined into 1 group, whereas apps from the iOS App Store were collected as a separate group (Figure 1).

The number of apps listed in the results of keyword search is unknown to customers in the iOS App Store, so we needed to confirm a range for selection. Moreover, we tested and found that the number of relevant apps appeared under each mental health–related keyword was less than 50 when counting downward from the top of the list. As searching through the 10 keywords, duplication was increasing. Therefore, we adopted the strategy of searching for the top 50 apps for each of the 10 keywords in every app store and then conducting deduplication. It is thus very likely to exhaust the relevant mental health apps in the China market.

### Selection of Mental Health Apps

All preselected apps were downloaded, installed, and registered on an iPhone 8s Plus and a Huawei P30 Pro for further selection. We accessed each app to further examine whether it was adequate for feature analysis and quality assessment with regard to the inclusion and exclusion criteria (Textbox 1). After removing the inadequate and duplicate apps, the remaining apps were combined for data extraction, analysis, and quality evaluation (Figure 1).

**Inclusion and exclusion criteria for mental health app selection.**

Inclusion criteria

Mental health apps would be included if they were:

- accessible;
- targeted at the individual mental health consumer, excluding mental health professionals and companies;
- usable;
- focused on relevant contents for people seeking professional assistance; and
- developed in Chinese or has a Chinese version.

Exclusion criteria

Mental health apps would be excluded if they were:

- not accessible (requiring enterprise/school identification to access);
- end products for doctors or commercial enterprises;
- not usable due to technical errors;
- focused on irrelevant contents such as entertainment and gaming, training and education, social and communication, business and commercial, romance and relationship, and e-books, which are irrelevant to mental health service; and
- duplication of apps with identical internal design or contents in the market.

### Data Extraction and Analysis

A table sheet was generated and saved in a Microsoft Excel file with specific variables to record the basic information and potential features of different aspects of each of the included apps. We extracted the data of apps regarding the themes and contents of mental health–informative articles (emotion control, depression, anxiety, stress management, and familial relationship), self-assessment of mental health status (different self-rating scales testing the level of depression, anxiety, autism, sleep quality, and personality), counseling services (types of counselors and ways of communication), mental health courses, and meditation. Data calculation and analysis were performed using SAS 9.4 on Microsoft Windows.

### Quality Evaluation Using Mobile App Rating Scale App Quality Criteria

The quality of included apps was evaluated using Mobile App Rating Scale (MARS) [31]. MARS contains 23 individual items sorted into the following 5 criteria categories: engagement, functionality, aesthetics, information, and subjective qualities. Each was rated by 2 independent investigators. The score of each individual item was rated from 1 to 5 points (1: inadequate, 2: poor, 3: acceptable, 4: good, and 5: excellent). Mean scores were calculated for the following 4 objective quality criteria: engagement, functionality, aesthetics, and information. In addition, a mean total score was calculated across the 4 objective categories. The 2 investigators were trained to use MARS by scoring a small group of apps individually and adjusting for discrepancies in understanding of the criteria. Disagreements were resolved by thorough discussions between the 2 investigators. We did not use the MARS app classification because of the fact that all the apps included were classified as mental health apps, and our focus was on app quality evaluation.

## Results

### Basic Information of Included Mental Health Apps

In total, 997 preselected mental health apps from Android and iOS markets were found through keyword search in the preselection (Figure 1). We found 327 relevant mental health apps from the iOS App Store, 268 apps from 360 Mobile Assistant, 276 apps from Baidu Mobile Assistant, and 126 from Tencent MyApp. Through further selection with respect to the inclusion and exclusion criteria, 63 unique apps were included for quality assessment and in-depth analyses (Figure 1).

Of the included apps, 78% (49/63) were developed by commercial entities providing mental health services, for example, Web-based mental health articles, psychological counseling, and mental health status assessment by self-rating scales. There were also 17% (11/63) apps developed by specialized psychiatric hospitals or psychological counseling agencies, featured by relevant services including appointment booking and consultation, for individual patients or clients of the entity. The rest were 2 apps provided by government or district-level Centers for Disease Control and Prevention (CDC) in Beijing, and 1 app developed for the students of Chongqing University.

### Features Built in Mental Health Apps

According to the findings above, the mental health apps in China were aimed to provide as broad range of mental health services as possible to nonspecific users. We only found 1 app purely for communication between depression patients (*Snail/ 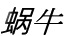
*). No apps were found purely for other common mental disorders, for examples, substance abuse and anxiety. Simultaneously, there has not been an app designed for the BPHS delivery in China that involves extensive procedures for the management of patients with severe mental disorders, including patient identification and registration, home visits, and drug dissemination [10]. Therefore, the following features were summarized with respect to the detailed services provided by the mental health apps found in the China market: the common features found in the mental health apps included were mental health education (67%, 42/63) either by informative articles or relevant courses, counseling services (65%, 41/63), self-assessment of mental health status (44%, 28/63), and question and answer (Q&A) module (40%, 25/63) providing quick answer to mental health–relevant questions that were available to all users. Other features included meditation (11%, 7/63) and self-management tools (16%, 10/63) such as drug description and intake alert and hotline for general mental health inquiries.

#### Mental Health Education

The mental health education component in the mental health apps can be accessed by either reading informative articles (38/63, 60%) or taking relevant courses (23/63, 37%). Informative articles addressed the causes, risk factors, symptoms, and coping skills of common psychological disorders or conditions, for instance, depression (33/63, 52%), anxiety (25/63, 40%), and emotion coping (34/63, 54%). For the apps providing mental health–related courses, 18 apps offered lectures with slides or recordings on the concise scientific knowledge of common mental disorders. In addition, 7 apps contained courses that were designed based on verified psychological therapy or training, for example, cognitive behavioral therapy (CBT) [32]. In total, 14 apps provided free courses. For paid courses, fees ranged largely according to different types across the apps.

#### Mental Health Counseling Services

Mental health counseling services were an essential feature in the included apps. A total of 80% (33/41) of the counseling services in these apps were provided by mental health professionals with verification. Counselors were verified by obtaining personally identifiable information or photocopy of their Counselor Certificate, and clinical psychiatrists were verified by obtaining the following information: name, title, and the hospital they work with. The users can purchase one-to-one counseling sessions through the apps. Web-based counseling sessions were a common method, including audio phone calls and video connection, available in 90% (37/41) of apps with the feature. Users can also write reviews and rate the counseling service, which was available to other app users. The fee for an mental health counseling session varied from less than 50 *yuan* to over 1000 *yuan* per hour.

#### Self-Assessment of Mental Health Status

Self-assessment of mental health status was conducted through self-rating scales. There were 28 apps providing at least 1 verified scale for depression or anxiety. Zung Self-rating Scale for Depression (21/28, 75%) and Zung Self-Rating Scale for Anxiety (SAS; 22/28, 79%) were the most frequently used tests for depression and anxiety detection, respectively [33,34]. Other scales used for depression self-diagnosis included Symptom Checklist 90 (15/28, 54%), Beck Depression Inventory (10/28, 36%), and Patient Health Questionnaire (PHQ) 9 (8/28, 29%) [35,36]. Scales for anxiety assessment also included Generalized Anxiety Disorder Screener (GAD-7; 4/28, 14%) and Beck Anxiety Inventory (6/28, 21%) [37,38]. Moreover, half (14/28, 50%) of the apps provided validated self-rating scales on sleep quality and somatization symptoms, including Athens Insomnia Scale and PHQ-15 [39,40]. In total, 89% of these apps (25/28) provided results for the self-rating scales.

#### Other Features of Mental Health Apps

Q&A section was available in 40% (25/63) of all the included mental health apps. Users can post questions relevant to mental health issues. Both mental health professionals and general users can respond to the questions, and all answers were open to be viewed by all users. Self-management–related features included tools for self-monitoring and management, such as mood diary, medication record, and reminder. There were also 11% apps (7/63) designed with modules on meditation training for everyday practice that aimed to improve stress coping, emotion regulation, and sleep quality.

### Quality Assessment Scores by Mobile App Rating Scale

The mean overall score of MARS was 2.96 for the total of 63 mental health apps (Table 1). Engagement was rated lowest of the 4 objective app quality criteria, which was 2.72. The highest was the mean function score, which was 0.53 higher than engagement. The aesthetics and information were similar, and both did not exceed the mean overall score.

In total, 56 apps can be classified as *acceptable*, and 8 apps as *good* according to the mean overall MARS scores given by the criteria (Table 2). However, there were still 7 apps rated as *poor* quality. For apps in the engagement category, 42 apps were classified into *acceptable* or above, whereas as high as 21 apps were classified into the *poor* group. The aesthetics category was similar to the mean overall group; the majority (55/63, 87%) of apps were *acceptable* or above, leaving 8 apps in the *poor* group. In the information category, 81% of the apps were *acceptable* or above, but still 12 apps were in the *poor* group. The function category was, in general, rated higher, given that 97% of all apps were classified into *acceptable* or *good* groups.

**Table 1:** Mean Mobile App Rating Scale scores of mental health apps (N=63).

Mobile App Rating Scale category	Minimum	Maximum	Mean (SD)
Engagement	1.8	4.0	2.72 (0.54)
Function	2.3	4.3	3.25 (0.06)
Aesthetics	2.0	4.0	2.99 (0.06)
Information	2.0	4.0	2.88 (0.54)
Overall	2.1	4.0	2.96 (0.40)

**Table 2:** Classification of mental health apps according to the mean Mobile App Rating Scale scores (N=63).

Mobile App Rating Scale category	Inadequate, n (%)	Poor, n (%)	Acceptable, n (%)	Good, n (%)	Excellent, n (%)
Engagement	—^a^	21 (33)	36 (57)	6 (10)	—
Function	—	2 (3)	38 (60)	23 (37)	—
Aesthetics	—	8 (13)	44 (70)	11 (17)	—
Information	—	12 (19)	42 (67)	9 (14)	—
Overall	—	7 (11)	48 (76)	8 (13)	—

^a^No app fell into this category.

## Discussion

### Summary and Discussion of Major Findings

Through the investigation into 63 of 997 mental health apps from iOS and 3 Android markets in China, major features and characteristics have been summarized from informative details. Features, including mental health education, counseling services, self-assessment of mental health status, and Q&A section, were commonly found in the apps across stores. The apps played the role of a platform gathering the users and the mental health professionals, allowing services to be delivered through the innovative platform. Moreover, the mean of overall MARS scores has shown that the quality was acceptable for the mental health apps. Quality scores of the 4 objective categories of MARS, engagement, aesthetics, function, and information, were similar to the overall rating. However, the apps, in general, rated better in the function category than others.

The rise of mental health apps in the China market might be because of deficient knowledge and negative perception toward mental disorders [12,41]. It has been a cultural stigma that mental illnesses in China are often considered as abnormal, and patients with mental disorders are shamed, especially in less developed rural area [10,41]. Therefore, people more tend to refer mental issues to physical symptoms, which may prevent people from actively seeking professional help [10]. Mobile health technologies, such as mental health apps, have the potential to protect patients’ privacy and reduce the impact of social stigma toward mental illnesses.

There were common features of mental health apps in China compared with apps in the global market, which include real-time communication through Web-based services and mental health education [27]. From the analyses of this study, most of the mental health apps provided Web-based counseling services. People can make real-time communication with their preferred mental health professionals in terms of mental health inquiries. Moreover, mental health information offered by the app can be an efficient tool to improve mental health literacy [42]. On the other side, differences were also found between mental health apps in China and global market. Apps in global market were commonly seen to be designed for a specific mental disorder or condition, for example, anxiety and depression [43,44]. Contrastingly, mental health apps in China were very likely to include information and services for a broad range of mental disorders or conditions in 1 single app. They often included a group of features in 1 app, such as counseling services, self-tests, and other mental health education on a broad range of psychological issues, instead of dealing with 1 particular disorder.

Moreover, most of the apps included in this study were developed by commercial entities for commercial purpose. Public health impact has hardly been stressed. More apps are expected to pay more attention to the common advantage from the public health perspective. For example, the patient management of severe mental disorders is a critical component of the BPHS in China, together with health records creation for every resident, health education, immunization, chronic disease (hypertension and diabetes) management, and so forth [11]. In China, BPHS has been provided through a laddered system of county hospital and CDC, township health care centers, and village clinics [11]. The frontline community health care workerss, who are more often called village doctors working at village clinics in rural China, have critical workload of patient finding and registration, home visit, and drug dissemination [5,20,45,46]. However, no mental health app was found to assist in such public health care services. Innovative health technologies are promising in mental health care practice in community and the whole mental health system of the country.

### Implications From Features of the Mental Health Apps

The popularity of counseling services reflects the increasing demand in mental health interventions of the general population. The apps act as a platform bridging the gap between mental health professionals and the app users, rather than directly engage in interventions for patients with the need of mental health care. Therefore, the mental health apps in China did not involve much core psychological methodologies but more incorporate functions of coordination and communication.

Web-based counseling outperforms traditional face-to-face counseling in a few ways. One aspect is that it allows reorganization of psychiatric resources, especially for available professionals. This can indeed benefit the Chinese population that is facing unbalanced and limited resources in mental health care [9]. However, concerns have also been raised regarding the quality of service provided by those psychological professionals. There is no standardized guidelines or official regulations for conducting Web-based counseling [47,48]. Moreover, it would be difficult for the mental health apps, as commercial entities, to effectively supervise counselors for their service.

Mental health apps can promote mental health education to lay public by offering informative articles and relevant courses. Low mental health literacy has long been considered as a major obstruction in help-seeking and treatment delivery for people with mental disorders in China [42,49]. However, quality concern has also been raised toward the mental health educational materials provided by mental health apps. It is necessary to establish more official guidance and promotion to improve mental health literacy.

Only 2 apps (*Alpha psychology/
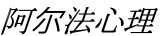
* and *Jianxinjiayuan/ 
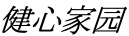
*) were found to be based on formal psychotherapeutic techniques of CBT [32]. CBT has been found effective in a variety of mental disorders and conditions, including affective disorders, psychosis, gambling behavior, and substance abuse [50-53]. Many apps from global market were also designed to incorporate the core techniques of CBT to elicit a cognitive and behavioral change of mental disorder and conditions, such as substance abuse and affective disorders [26,54]. More mental health techniques and psychotherapy components, including techniques used in CBT, are expected to be involved in designing the feature modules.

Self-rating scales of common mental disorders, such as depression and anxiety, were prevalent in mental health apps. Verified self-rating scales were convenient tools for quick assessment. Some apps even provided multiple rating scales for 1 disorder that allows users to compare GAD-7 and SAS for anxiety testing. However, there were drawbacks in using the Web-based version of self-rating scales. These include errors in the questions, as well as misunderstanding and misinterpretation of the test results. Most self-rating scales were written originally in English and translated directly into Chinese; therefore, the questions were asked in the way more suitable for Western lifestyle and culture [55]. Translation errors and discrepancies always occurred. Moreover, there are special requirements for doing self-tests; for instance, some questions may require intuitive answers, but some may involve precise recalling. Therefore, it is better to have the presence of a trained mental health professional when scoring the scales. In addition, lay users may feel to be overlabeled for a mental disorder, and unnecessary panic can occur [27,56].

In addition, features of self-management were found in some apps, including mood diary, drug description, and medication reminder. Those features can be practical tools to support regular treatment for clinical patients already diagnosed with mental disorders [20]. However, they were overall less *smart* and not relevant to mental health, for example, a drug reminder is a simple clock. Considering gaps currently in the mental health services in China, the developers might put more efforts to combine the potential of smart technologies and mental health relevance [5]. Meditation is another growing aspect of mental health app features. The apps provided simple guidance by recorded audio clips. Therefore, it is 1-direction demonstration, and no feedback was required from the users. However, the users did not have the opportunity to correct or improve in everyday practice.

### Quality Evaluation by Mobile App Rating Scale

This study used MARS quality criteria to evaluate the 63 included mental health apps [31]. Both mean overall score and individual score of the 4 objective categories had shown that the majority of the apps were *acceptable*, which was the middle of the 5-point scale. The quality did not vary much across the 4 trajectories of engagement, aesthetics, function, and information, but the function category was rated to be slightly higher. The mean scores of MARS suggested that there would be space for improvement in the above trajectories. For instance, mental health apps can pay more attention to the visual appeal, content engagement, and display of app-relevant information in the market.

### Strengths and Limitations

This is the first academic review on the characteristics and common features of mental health apps in China. Limitations might include that we searched mental health apps by keyword, following the default algorithm of each app market. This is to simulate the real-world situation when mobile phone users search for mental health apps in common app markets. Moreover, built-in features that required in-app purchase to be accessed were out of the scope of analyses. For example, one should pay for a counseling session to communicate with a counselor in an app with that feature. However, the analyses of this study were purely descriptive. Further research on experiences of the paid features is expected by qualitative study in the future.

### Conclusions

In conclusion, this study has systematically reviewed and investigated the built-in features of mental health apps in current China market. Moreover, the quality of the apps was also evaluated using valid assessment criteria. Overall, the area of mental health–related innovative technologies in China is experiencing fast development, and appropriate guidance would be beneficial to the growth of the field.

## Abbreviations

BPHS: Basic Public Health Service
CBT: cognitive behavioral therapy
CDC: Centers for Disease Control and Prevention
GAD: Generalized Anxiety Disorder Screener
MARS: Mobile App Rating Scale
PHQ: Patient Health Questionnaire
Q&A: question and answer
SAS: Self-Rating Scale for Anxiety
